# The safety climate in primary care (SAP-C) study: study protocol for a randomised controlled feasibility study

**DOI:** 10.1186/s40814-016-0096-5

**Published:** 2016-09-16

**Authors:** Sinéad Lydon, Margaret E. Cupples, Nigel Hart, Andrew W. Murphy, Aileen Faherty, Paul O’Connor

**Affiliations:** 1 Discipline of General Practice, National University of Ireland, Galway, Ireland; 2 Department of General Practice and Primary Care, School of Medicine, Dentistry and Biomedical Sciences, Queen’s University, Belfast, UK; 3 UKCRC Centre of Excellence for Public Health Research (NI), Queen’s University, Belfast, UK

**Keywords:** Patient safety, Safety climate, Safety culture, Primary care, General practice, Intervention, Audit, Questionnaire

## Abstract

**Background:**

Research on patient safety has focused largely on secondary care settings, and there is a dearth of knowledge relating to safety culture or climate, and safety climate improvement strategies, in the context of primary care. This is problematic given the high rates of usage of primary care services and the myriad of opportunities for clinical errors daily. The current research programme aimed to assess the effectiveness of an intervention derived from the Scottish Patient Safety Programme in Primary Care. The intervention consists of safety climate measurement and feedback and patient chart audit using the trigger review method. The purpose of this paper is to describe the background to this research and to present the methodology of this feasibility study in preparation for a future definitive RCT.

**Methods:**

The SAP-C study is a feasibility study employing a randomised controlled pretest-posttest design that will be conducted in 10 general practices in the Republic of Ireland and Northern Ireland. Five practices will receive the safety climate intervention over a 9-month period. The five practices in the control group will continue care as usual but will complete the GP-SafeQuest safety climate questionnaire at baseline (month 1) and at the terminus of the intervention (month 9). The outcomes of the study include process evaluation metrics (i.e. rates of participant recruitment and retention, rates of completion of safety climate measures, qualitative data regarding participants’ perceptions of the intervention’s potential efficacy, acceptability, and sustainability), patient safety culture in intervention and control group practices at posttest, and instances of undetected patient harm identified through patient chart audit using the trigger review method.

**Discussion:**

The planned study investigates an intervention to improve safety climate in Irish primary care settings. The resulting data may inform our knowledge of the frequency of undetected patient safety incidents in primary care, may contribute to improved patient safety practices in primary care settings, and may inform future research on patient safety improvement initiatives.

**Electronic supplementary material:**

The online version of this article (doi:10.1186/s40814-016-0096-5) contains supplementary material, which is available to authorized users.

## Background

Safety culture can be defined as “the shared values, attitudes, and behaviour of all staff in health facilities in regard to giving safety priority over efficiency, improving care provider communication and collaboration, and creating a system that learns about and learns from errors and problems” [1]. The terms safety culture and safety climate are often used interchangeably in the literature. However, it is possible to distinguish between the constructs; safety culture represents the more stable and enduring traits of the organisation and has been likened to personality, while safety climate is a measurable snapshot of an underlying safety culture at a particular period of time [2–4]. Research in secondary care settings has identified an association between safety culture or climate and adverse events [5, 6], mortality [7], and error reporting [8].

While research assessing, and interventions to improve, patient safety in hospital settings has become increasingly commonplace over the past 20 years [9], the study of patient safety in primary care settings has lagged behind [3, 10–12]. This slower uptake may relate to the perception of primary care delivery as a relatively low-risk endeavour far less likely to result in patient harm than secondary care services given the infrequency of adverse events in primary care and the lesser use of technology in the delivery of services [10]. However, both patient factors [13], such as increasingly complex co-morbidities and polypharmacy, and practice factors, such as large volumes of patients and increasing time pressure and workload [14], have resulted in a growing complexity of practice for general practitioners and an increased potential for clinical errors in primary care.

In the United Kingdom (UK), 85 % of patient contact with the National Health Service (NHS) is in primary care settings, 750,000 persons see their general practitioner on any given day, and 70 % of all prescriptions are written by primary care practitioners [10]. While reports of safety climate in UK primary care settings are largely positive [15], there is a discrepancy in the report of “managerial” staff members and other employees that may indicate an overestimation of positive safety climate in these settings. This suggestion is supported by research which has found that there are between 5 and 80 patient safety incidents, or errors, made per 100,000 primary care consultations [16]. Other researchers have estimated that 2.2 % of all patient consultations in primary care result in a patient safety incident [17]. The UK-based National Patient Safety Agency has defined patient safety incidents as “any unintended or unexpected incident which could have or did lead to harm for one or more patients receiving [medical] care” [18]. Patient safety incidents may therefore include issues in investigation, diagnosing, prescribing, information handling, and doctor-patient communication [10, 15]. The outcomes of patient safety incidents vary; research suggests that 50 % have no ill consequences, 20 % result in delays in diagnoses, 10 % result in patient distress, and 20 % have serious consequences for the patient’s health [19]. British primary care physicians have noted a number of impediments to monitoring patient safety in their workplaces including a lack of access to pertinent data or uncertainty regarding the metrics of patient safety that should be monitored, a lack of clarity regarding policies and procedures for assessing patient safety and addressing incidents of harm, and a lack of clarity surrounding whose responsibility is the monitoring of patient care [20].

Data such as these have prompted the World Health Organisation to note the pressing need to study and address patient safety in primary care settings [21]. In secondary care settings, interventions to improve safety culture have included leadership walk rounds, educational programmes, team training, simulation-based training programmes, unit-based safety programmes, and multi-component organisational interventions [4], with varying degrees of evidence available to support these approaches. However, a systematic review [3] of interventions to improve safety culture in primary care identified only two published interventional studies. One of these studies evaluated the impact of an electronic medical record system while the other assessed the utility of workshops on risk management and significant event analysis and practice-specific quality improvement activities. These studies reported positive outcomes, but interpretation of effects is hampered by methodological issues that precluded the derivation of recommendations [3].

The current research programme seeks to evaluate the intervention developed as part of the Scottish Patient Safety Programme in Primary Care (SPSP-PC) [11] in the context of primary care settings in the Republic of Ireland and Northern Ireland. Tested over a 2-year period, the SPSP-PC programme was launched nationally in Scotland in 2013 and comprises the first known comprehensive and coordinated attempt to improve patient safety in primary care settings in any country [11]. The intervention consists of the use of the GP-SafeQuest safety climate measure [22] and the use of a trigger review method (TRM) [23] for identifying undetected instances of patient harm from patient charts, as diagnostic learning tools. Recent data indicate that 90 % of primary care practices in Scotland have adopted the intervention, and 83 % have reported that the intervention has allowed them to make changes within their practice that have resulted in an improved quality of, and safer, patient care [11]. General practitioner feedback on the intervention’s acceptability, feasibility, and utility in the Scottish context has been predominantly positive [24]. Further, data support the utility of the TRM process, indicating that 14.1 % of 13,351 records reviewed by Scottish general practitioners contain a previously undetected patient safety incident and that improvement actions implemented as a result were described in over 85 % of the summary reports resulting from the TRM audits [25].

### Objectives

In spite of the high costs of medical errors in Northern Ireland [26] and the Republic of Ireland [27], there is a notable dearth of published research investigating interventions to improve safety climate or culture in primary care settings [3], particularly in countries outside of the USA, UK, and Australia [28]. The planned study is thus designed to address this research gap. The primary objective of this study is to inform the planning of a definitive randomised controlled trial by providing evidence on whether the intervention is feasible, useful, and sustainable in the Irish context, by determining the rates of recruitment and completion of the outcome measures and by exploring the acceptability of the intervention to study participants. A secondary objective is to allow an estimate of an appropriate sample size to be calculated to ensure that a possible future definitive trial is adequately powered. Therefore, while the outcomes of the intervention for the safety climate of the participating practices are of interest, the evaluation of the process of its implementation is the primary aim.

## Methods/design

### Context

General practices in Ireland are operated independently of the state healthcare system. General practitioners may work in single practices, group practices, and primary care centres or health centres. Patients typically pay privately to attend a general practitioner, but some patients with special circumstances (e.g. chronic health conditions, disability, unemployment, advanced age) may possess a medical card that allows them to attend their general practitioner without paying a fee. These general practitioners receive reimbursement from the Health Service Executive for these patients.

General practices in Northern Ireland fall under the remit of the NHS.

### Design and setting

The SAP-C study is a feasibility study [29] for a future randomised controlled trial. The study uses a randomised controlled design. The study will be conducted in eight practices in the Republic of Ireland and two practices in Northern Ireland.

### Practices selection and randomisation

In the Republic of Ireland, practices will be recruited through the Western Research Network (WestREN) [30], an Irish general practice research network. Practices will be stratified according to size (i.e. small or large) and location (i.e. urban or rural) so that a diverse range of practices, reflective of the national profile, can be invited to participate in the research programme. In total, eight practices reflective of the national profile will be chosen and invited to participate. In Northern Ireland, two practices will be recruited through the Northern Ireland Clinical Research Network Primary Care Group, with a similar size and location. The choice will be pragmatic accounting for the limited resources available to this feasibility study (i.e. travel distance will be considered). The invitation letter/email will be sent to the principal general practitioner(s) at the practices initially, and once consent to participate has been obtained from the principal general practitioners, then, the other staff members will be invited to take part (see Additional file 1). If we fail to obtain the required number of practices to participate, then additional practices will be invited until the desired number of practices has been recruited. In total, eight practices will be recruited in the Republic of Ireland.

This study will employ simple randomisation. In both Northern Ireland and the Republic of Ireland, an equal number of participating practices will be randomised to either the intervention or control group using online randomisation software by a researcher external to the research group. Given the nature of the intervention to be delivered, blinding is not feasible.

### Procedure

**Fig. 1:**
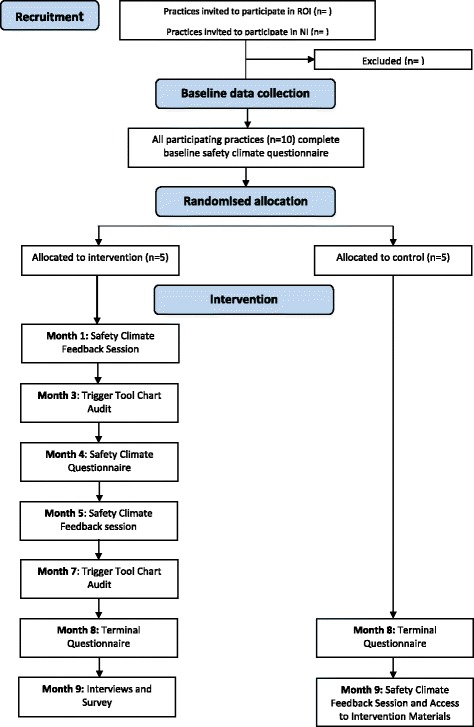
Flow diagram of the SAP-C feasibility study

Figure 1 provides an overview of the intervention and control group activities and the associated timeline.

#### Intervention group

The SPSP-PC intervention to be utilised in this study is comprised of two components: (1) safety climate measurement and feedback using the GP-SafeQuest and (2) patient chart audit using the trigger review method. In this study, safety climate will be measured using the 30-item GP-SafeQuest measure for primary care [22], used previously in the SPSP-PC study and elsewhere [15], at three time points (i.e. baseline, study midpoint, and study terminus; see Fig. 1). Subject-generated identification codes will be used to track respondents across the three data collection points. The GP-SafeQuest has been demonstrated to be a valid and reliable measure of perceptions of the prevailing safety climate in primary care settings [22] and consists of five subscales:

1. Leadership (6 items)
2. Teamwork (7 items)
3. Communication (5 items)
4. Workload (4 items)
5. Safety systems (8 items)

Items are rated using a 7-point rating scale ranging from 1 (not at all) to 7 (to a very great extent). Questions soliciting information on demographics (i.e. position in practice, years of work experience, and gender) are also included. All staff members (i.e. general practitioners, nurses, administrative staff) at each participating practice assigned to the intervention group will be invited to complete this questionnaire. Following the completion of the first and second questionnaires, each intervention group practice will receive individualised practice-level feedback on safety climate during an in-practice lunchtime workshop and a specific report. Simple descriptive statistics and illustrative diagrams will be used to facilitate practice staff members’ understanding of the data. Further, practices will be presented with data showing their practices’ safety climate scores compared to the anonymised data from other practices participating in this study. The ultimate purpose of the feedback session is to better inform participants about the safety climate in their practice, to promote reflection and learning, and to provide suggestions for strategies that may result in improved safety climate.

One general practitioner from each practice will also be asked to complete a patient chart audit at 3 and 7 months using a specialised trigger tool [23]. This trigger tool consists of ten triggers:

1. Timing of consultation
2. Place of consultation
3. Frequency of consultation
4. Changes to medication
5. Adverse drug events/allergies
6. New clinical read code
7. Abnormal blood results
8. Out-of-hours and/or A&E
9. Hospital admission/discharge
10. >1 outpatient appointments in the past year

The TRM’s efficacy in identifying undetected patient harm and/or existing system hazards has been demonstrated in a number of research studies [5–23, 31]. General practitioners will attend a 2-h training workshop on applying the TRM in advance of conducting the audit. The training workshop will be modelled on the TRM educational intervention developed by McKay and colleagues [24]. Training comprises a brief PowerPoint presentation about the TRM and its role in safety improvement in primary care settings, a patient safety quiz, a group work exercise to ensure participants’ understanding of the key constructs being assessed and studied, training in TRM (i.e. step by step guidance, viewing worked examples, viewing sample summary reports), practice applying the TRM to simulated records, and a take-home exercise allowing participants to use the TRM with their own patient charts. For both audits conducted as part of the intervention, a general practitioner in each intervention group practice will apply the trigger tool [23, 31] to a total of 20 records from a high-risk patient group (>75 years of age) randomly selected from patients that have attended the practice over the past 3 months. The review is a two-phase process. Firstly, the records are reviewed in order to detect whether they contain a trigger. If a trigger is found in a patient’s record, then it should be reviewed in more detail to determine whether the patient experienced any harm. For the purpose of this research study, harm is defined as “anything that happens as a result of interaction with health services (environment, workers, treatment) that you would not want to happen to you or your relatives” [32]. If no harm is detected, or the reviewer is unsure, they should not record the incident. If harm is detected, the reviewer should classify the severity of the harm and determine whether it was avoidable. This audit should prompt reflection, learning, and/or further action within the general practice. The reviewer should use the process to identify possible immediate actions based upon the findings of the audit (e.g. intervene to address any issue(s) with specific patients or to tackle any system hazards that are identified, identify a targeted intervention to prevent future occurrences). Moreover, members of the practice will also be encouraged to discuss, with their colleagues, any recent events/cases that may not have been identified in the chart review but that may have implications for patient safety. Any actions to be taken on the basis of the chart audit or in-practice discussion are to be decided by the practice. The reviewer will also share their findings with the practice team in order to foster collective learning. The practices will only be asked to share summary information about the review with the researchers. This information will include the number of chart reviews, which triggers were identified, number of incidents of harm and level of severity, whether the harm was preventable, and a brief description of any measures that were taken as a result of the review.

Following the conclusion of the intervention, general practitioners in the intervention group will be asked to complete a brief intervention satisfaction survey in order to provide the researchers with information on whether participants thought the intervention was effective, useful, and sustainable within Irish general practice settings. Further, at least three general practitioners in each intervention group practice will be asked to take part in a semi-structured interview so that their experiences with, and perceptions of, the intervention can be explored.

#### Control group

As can be seen in Fig. 1, control group activities are notably fewer than those described for the intervention group. All individuals working in the control group practices (e.g. general practitioners, nurses, administrative staff) will be invited to complete the GP-SafeQuest [22] at baseline and at the study’s terminus. However, unlike in the intervention group, the control group practices will not receive feedback on the safety climate in their practice during the study. Following the conclusion of the study, these practices will receive feedback on their safety climate and will be provided with access to the study materials.

### Outcome measures

#### Process evaluation

As this study comprises a feasibility study for a future randomised controlled trial, the evaluation of the implementation process is the primary outcome. Quantitative data that will contribute to our understanding of this outcome include rates of recruitment and retention of participants and rates of completion of the safety climate measure. The data from the post-intervention semi-structured interviews, and brief intervention satisfaction surveys, conducted with participants in the intervention group will also yield data pertinent to this outcome. Both the survey and the interviews will allow for the probing of participants’ attitudes and perceptions of the intervention’s potential efficacy, utility, sustainability, and acceptability in the context of Irish primary care settings. Barriers to compliance with, or potential efficacy of, the intervention will also be examined with a view to adapting or modifying the intervention in a future randomised controlled trial for optimum efficacy.

#### Safety climate

In addition to functioning as a diagnostic learning tool, the GP-SafeQuest measure of safety climate employed in the current study will also be used to evaluate the impact of the intervention. For the intervention group practices, it will be possible to track changes in safety climate across the measurement time points (i.e. baseline, study midpoint, and study terminus). The comparison of safety climate at the study’s terminus (posttest time point) within intervention group practices and control group practices will also allow for an evaluation of the impact of the intervention. Finally, the effect sizes observed here (i.e. any change in mean safety climate scores as a result of the intervention or any difference in mean safety climate scores at posttest between the intervention and control groups) will also contribute to the planning of a future definitive trial. This knowledge of an observed effect size in the feasibility study will be considered along with the feasibility of detecting different levels of effect sizes and the real-world importance of the various effect sizes, when setting a desired effect size for the definitive study and determining an appropriate sample size.

#### Patient harm instances

A general practitioner from each intervention group practice will conduct two patient chart audits using the TRM over the course of the intervention period. A summary of the findings of each audit (i.e. triggers identified, number of instances of undetected patient harm identified, severity of each instance of harm detected, potential avoidability or preventability of each instance of harm detected, and description of action resulting from audit) will be provided to researchers. These data will provide an indicator of the potential efficacy of the TRM in identifying undetected instances of patient harm and its usefulness.

### Data management and analysis

As this is a feasibility study, statistical analyses will be primarily descriptive, as recommended in best practice guides for pilot and feasibility studies [29, 32]. A descriptive report of the mean change in the scores on each of the scales of the GP-SafeQuest pre- and post- intervention for the intervention and control groups will be produced alongside a measure of the range of these scores. Each participating GP practice will be given a code. The code key will be encrypted and stored separately from the database of questionnaire responses. As the individual respondents will be tracked using subject-generated identification codes, the individual level data will be anonymous.

The qualitative data emerging from the post-intervention semi-structured interviews will be analysed using the framework method for qualitative data analysis in accordance with best practice [33]. Each interview participant will be given a code. The code key will be encrypted and stored separately from the interviews. Following transcription, the interview recordings will be destroyed. Both the questionnaire and interview transcripts will be stored on a password-protected computer. The data will be stored at NUI Galway, and only the researchers will have access.

### Dissemination policy

The findings from the study will be published as a journal paper and presented at an international primary care conference. The findings will also be disseminated via the internet at www.primarycaretrials.ie and through social media.

## Discussion

This study will investigate the impact of an intervention, derived from the SPSP-PC, on the safety climate of primary care practices. We will investigate if this intervention, comprising safety climate measurement and feedback using the GP-SafeQuest and patient chart audit using the trigger review method, results in changes in safety climate among intervention group general practices as compared to the control group and aids with the detection and remediation of instances of patient harm. As this is a feasibility study, process evaluation will form the core outcome measure allowing for an assessment of the potential efficacy, utility, acceptability, and sustainability of the intervention in Irish primary care settings and the anticipation of potential pitfalls of a future randomised controlled trial (e.g. low recruitment rates, attrition, low rates of completion of measures). Given the high usage of primary care services, and the rates of errors or instances of undetected patient harm reported in primary care in other countries [16, 17], it is anticipated that the intervention in the current study will provide useful data surrounding the prevalence of undetected patient harm in Irish primary care and the safety climate of Irish general practices.

There are a number of strengths of this study. There is a noted gap in the research literature surrounding the assessment of, and intervention to improve, safety climate in primary care settings [3, 10–12], particularly in countries outside of the UK, USA, and Australia [28, 34]. The current study will assess, and intervene upon, safety climate in Irish primary care settings providing novel and interesting data. Further, the study involves the delivery of an intervention for which initial evaluative data emerging from Scotland have been positive [11] but which requires additional empirical evaluation and validation. However, the likelihood of intervention success is improved by the validation of the individual interventional components (i.e. the safety climate measure and the TRM) in previous research studies [15, 22–25, 35]. Finally, the use of a mixed methods approach may also be considered a strength of the study. Verbakel and colleagues [3] have previously noted the complexity of multi-component patient safety interventions designed for implementation in primary care settings. A mixed methods approach is therefore suitable in order to capture participant experiences and valuable qualitative data to contribute to process evaluation and inform a future, larger trial of the intervention.

There are also a number of limitations to the planned study. First, there is a potential for selection bias to occur as practices that are invited to participate either opt-in or opt-out. In this way, practices in which there is a greater focus on patient safety, and consequently an increased motivation to implement interventions to improve patient safety, may be more likely to choose to partake in the proposed study and this may impact upon intervention outcomes. Second, there is also a reliance on self-report in the current study (i.e. self-report of safety climate, self-report of incidents of patient harm identified) rather than objective data. However, due to the nature of the variables under measurement (i.e. perceptions of safety climate, incidents of harm detectable only through the review of confidential patient information), it is not feasible to plan to collect more objective data.

The planned research programme will contribute to existing knowledge by evaluating an emerging intervention for patient safety in the context of Irish primary care settings. The resulting data may inform our knowledge of the frequency of patient safety incidents in primary care and contribute to an improved standard of care delivered by general practitioners.

## Additional file

Additional file 1: Invitation letters, participant information sheets, and statement of consent forms. (DOCX 45 kb)

## Abbreviations

NHS: National Health Service
SAP-C: Safety climate in primary care
SPSP-PC: Scottish Patient Safety Programme in Primary Care
TRM: Trigger review method
UK: United Kingdom
WestREN: Western Research Network
