# Supplementary material for: The safety climate in primary care (SAP-C) study: study protocol for a randomised controlled feasibility study
Source: Pilot Feasibility Stud. 2016 Sep 16;2:56. doi: 10.1186/s40814-016-0096-5 (PMC5154125; doi:10.1186/s40814-016-0096-5)
Supplement: Additional file 1: — Invitation letters, participant information sheets, and statement of consent forms. (DOCX 45 kb) [file 40814_2016_96_MOESM1_ESM.docx]

**Invitation letters, Participant Information Sheets, and Statement of Consent Forms**

**Invitation Letter (#1) (Initial Invitation Letter for Principal GPs only)**

Dear General Practitioner,

As part of the Irish Primary Care Trials Network funded by the Health Research Board, your practice is being invited to participate in a Health Research Board funded study entitled ‘The SAP-C study- A feasibility study of the effectiveness of an intervention designed to improve Safety in Primary Care’. A large volume of work has examined the effectiveness of interventions to improve patient safety in the hospital setting but little research has considered patient safety in primary care settings.

**Study description**

The purpose of this research study is to evaluate an intervention intended to improve patient safety in primary care settings. The intervention consists of:

- the measurement (via a questionnaire) of safety climate of the practice;
- provision of individualised feedback to participating practices on their safety climate; and
- the completion of two chart audits by a member of the participating practices using a trigger checklist (the individual will be paid at locum rates to carry out the audits).

The impact of this intervention, adapted from the NHS Safer Primary Care Scotland model, upon safety climate will be assessed by comparing the outcomes of the intervention group to those of a control group who have not received the intervention. Practices who agree to participate will be randomly assigned to either the intervention group or a control group. The control group participants will be asked to complete a questionnaire pertaining to the safety climate of their practice at two timepoints.

**What are the advantages for my practice?**

- Participants assigned to the intervention group will have the opportunity to apply for Irish Medical Council continuing education credits for conducting the chart audit and attending in-practice teaching and feedback sessions on safety climate.
- Participants will receive feedback on the safety climate in their practice which may allow for improved patient safety.
- Participants assigned to the intervention group will receive payment at locum rates for completing the associated chart audit or attending training in conducting the chart audit.
- Participants will be entered into a prize draw on each occasion that they complete the safety climate questionnaire.

The enclosed participant information sheet provides comprehensive information on what participation in this study involves and the compensation that participants will receiving for taking part. Please read this information sheet carefully if you are interested in having your practice taking part. Participation is voluntary and anonymous. If you wish to take part having read the participant information sheet, please return a signed copy of the statement of consent form to the researchers via the self-addressed envelope provided. If you agree to have your practice take part then we will then contact you to arrange to distribute information sheets and consent forms to the other staff members so that they may decide whether or not they wish to be involved.

Please do not hesitate to contact [investigator] via phone or email if you need any further information, would like to discuss this study in person, or have any questions regarding this research study. Thank you for taking the time to read this.

Kind regards,

[Investigators’ details here]

**Participant Information Sheet (#1) (Principal GPs)**

**Introduction.** Your practice is being invited to participate in a Health Research Board funded study entitled ‘The SAP-C study- A feasibility study of the effectiveness of an intervention designed to improve Safety in Primary Care’

**Procedures.** Participating practices will be randomly assigned to either the intervention group or control group. The activities involved differ between the groups and are described below.

*Intervention group:* If you are assigned to the intervention group, staff members at your practice (you, other general practitioners, nurses, receptionists etc.) will be asked to complete a questionnaire concerning patient safety in your practice at three timepoints (commencement of intervention, mid-intervention, and at the conclusion of the intervention. Each questionnaire will take about 10 minutes to complete. Following this, the researchers will provide practice-specific feedback on the safety climate of your organisation at each of these timepoints. One general practitioner in each participating practice will also be asked to complete a patient chart audit at two timepoints and to provide summary information to the researchers on the outcomes of this audit (i.e., number of charts reviewed, number of incidents of concern identified etc.). This audit will be conducted using a special checklist that will be provided by the researchers. Finally, at the conclusion of the intervention, participating general practitioners will be asked to complete a brief intervention satisfaction survey and to take part in a short interview concerning your experiences during the intervention and to share your perceptions of the utility and feasibility of the intervention in the Irish primary care context.

*Control group:* If you are assigned to the control group, staff members at your practice (you, other general practitioners, nurses, receptionists etc.) will be asked to complete a questionnaire concerning patient safety in your practice at two timepoints. The questionnaire will take about 10 minutes to complete on each occasion. Following conclusion of the intervention, all materials relating to the intervention chart audit, and feedback on your practice’s safety climate, will be made available to you.

**Risks.** The potential risks of participating in this study are that you or other staff members may determine that safety practices in your general practice are less than optimal, or, if assigned to the intervention group, that the general practitioner responsible for conducting the chart audit may identify instances of patient harm. These risks are no greater than the risks associated with the chart audit and practice evaluation that should be conducted yearly to comply with the Irish College of General Practitioner’s continuing professional development guidelines. There is also a risk of a breach in confidentiality. However, all data that you provide will be coded in order to ensure that individual participants are not identifiable from the data and all data will be stored securely.

**Benefits.** Participation in this study may aid with the improvement of the safety practice of your general practice and may result in improved patient safety. The results of the study will also be used to inform further study of the safety climate of general practice in Ireland and may lead to improved patient outcomes nationally.

**Compensation.** All participants who complete the questionnaire will be entered into a draw to win one of several 250e vouchers at each timepoint. In the intervention group, the general practitioner responsible for conducting the patient chart audit will be financially compensated for the time involved at a locum rate. For general practitioners enrolled on the ICGP professional competence scheme, completion of the patient chart audit may be used to obtain CPD credits.

**Confidentiality.** All data collected will be confidential and will be stored securely. Data will be stored securely and participants will not be identifiable from the data.

**Voluntary Nature of the Study**. Any information that is obtained during the study will be kept confidential. Only the researchers will have access to the data. No information will allow responses to be linked to a particular individual. Participation is strictly voluntary. It can be withdrawn at any time without affecting your rights in any way.

**Points of Contact**. It is understood that should any questions or comments arise regarding this study, the co-investigator [contact details here] should be contacted.

### **‘The SAP-C study- A feasibility study of the effectiveness of an intervention designed to improve Safety in Primary Care**

### **STATEMENT OF CONSENT (#1)**

### I have read and understood the Participant Information Sheet associated with this research study. I have had the opportunity to ask questions. If I have asked questions, these have been answered to my satisfaction.

I understand that I don’t have to take part in this study and that I can opt out at any time. I understand that I don’t have to give a reason for opting out and I understand that opting out won’t affect my legal rights, future medical care or my job within the practice.

### I am aware of the potential risks of this research study.

**Storage and future use of information:** I give my permission for information collected about me to be stored or electronically processed for the purpose of scientific research and to be used in related studies or other studies in the future but only if the research is approved by a Research Ethics Committee.

**Statement of Consent**: I have read the information provided above. I have been given the opportunity to ask questions and have had enough time to consider the information. I understand that my participation is voluntary and that I am free to withdraw this at any stage, without giving any reason, without my legal rights being affected.

**Participation of other practice staff:** I give my permission for the researchers to contact other staff members within my practice to invite them to take part in this research study. These participants will receive a participation information sheet and statement of consent form in order to allow them to consider participating in this research study.

### Principal GP Name (Block Capitals):

Principal GP Signature:

Date:

**Invitation Letter (#2) (General Practitioners)**

Dear General Practitioner,

As part of the Irish Primary Care Trials Network, you are being invited to participate in a Health Research Board funded study entitled ‘The SAP-C study- A feasibility study of the effectiveness of an intervention designed to improve Safety in Primary Care’.

You are being invited to participate in this research study as the principal GP(s) in your practice have indicated their willingness for the practice staff members to take part if they choose to. The enclosed participant information sheet provides comprehensive information on what participation in this study involved. Please read this information sheet carefully if you are interested in taking part. Participation is voluntary and anonymous. If you wish to take part having read the participant information sheet, please return a signed copy of the statement of consent form to the researchers via the self-addressed envelope provided.

Patient safety is a core element of the eight domains of good professional practice as outlined by the Irish Medical Council. However, medical errors remain common and can result in considerable patient harm. A large volume of work has examined interventions to improve patient safety in the hospital setting but little research has considered patient safety in primary care settings, although the majority of consultations occur in these contexts. With increasing demands on general practitioners in terms of workload, time constraints, early discharges from hospital and polypharmacy, there is a significant potential for error that must be addressed.

The purpose of this research study is to evaluate an intervention intended to improve patient safety in primary care settings. The intervention consists of the measurement of safety climate in participating general practice settings (via questionnaire) and provision of individualised feedback to participating practices, and the completion of a chart audit by participating practices using a trigger checklist. The impact of this intervention upon safety climate will be assessed by comparing the outcomes of the intervention group to those of a control group who have not received constructive feedback on their practices’ safety climate and who have not conducted the chart audit. The intervention that will be implemented is adapted from the NHS Safer Primary Care Scotland model. The effects of the intervention will be measured by comparing the outcomes of those experiencing the intervention to those of a control group. Practices that participate will be randomly assigned to either the intervention or control groups.

Please do not hesitate to contact [co-investigator] via phone or email if you need any further information, would like to discuss this study in person, or have any questions regarding this research study. Thank you for taking the time to read this.

Kind regards,

[Investigators’ details here]

**Participant Information Sheet (#2) (General Practitioners)**

**Introduction.** You are being invited to participate in a Health Research Board funded study entitled ‘The SAP-C study- A feasibility study of the effectiveness of an intervention designed to improve Safety in Primary Care’

**Procedures.** Participating practices will be randomly assigned to either the intervention group or control group. The activities involved differ between the groups and are described below.

*Intervention group:* If your practice is assigned to the intervention group, staff members at your practice will be asked to complete a questionnaire concerning patient safety in your practice at three timepoints (commencement of intervention, mid-intervention, and at the conclusion of the intervention. Each questionnaire will take about 10 minutes to complete. Following this, the researchers will provide practice-specific feedback on the safety climate of your organisation at each of these timepoints. One general practitioner in each participating practice will also be asked to complete a patient chart audit at two timepoints and to provide summary information to the researchers on the outcomes of this audit (i.e., number of charts reviewed, number of incidents of concern identified etc.). This audit will be conducted using a special checklist that will be provided by the researchers. Finally, at the conclusion of the intervention, participating general practitioners will be asked to complete a brief intervention satisfaction survey and to take part in a short interview concerning your experiences during the intervention and to share your perceptions of the utility and feasibility of the intervention in the Irish primary care context.

*Control group:* If your practice is assigned to the control group, you will be asked to complete a questionnaire concerning patient safety in your practice at two timepoints. The questionnaire will take about 10 minutes to complete on each occasion. Following conclusion of the intervention, all materials relating to the intervention chart audit, and feedback on your practice’s safety climate, will be made available to you.

**Risks.** The potential risks of participating in this study are that you may determine that safety practices in your general practice are less than optimal, or that you may identify instances of patient harm if you conduct the patient chart audit. These risks are no greater than the risks associated with the chart audit and practice evaluation that should be conducted yearly to comply with the Irish College of General Practitioner’s continuing professional development guidelines. There is also a risk of a breach in confidentiality. However, all data that you provide will be coded in order to ensure that individual participants are not identifiable from the data and will be stored securely.

**Benefits.** Participation in this study may aid with the improvement of the safety practice of your general practice and may result in improved patient safety. The results of the study will also be used to inform further study of the safety climate of general practice in Ireland and may lead to improved patient outcomes nationally.

**Compensation.** Participants who complete the questionnaire will be entered into a prize draw to win one of several 250e vouchers at each questionnaire completion timepoint. The general practitioner who completes the patient chart audit will be compensated financially, at a locum rate, for the time required to complete the audit.

**Confidentiality.** All data collected will be confidential and will be stored securely. Data will be stored securely and participants will not be identifiable from the data.

**Voluntary Nature of the Study**. Any information that is obtained during the study will be kept confidential. Only the researchers will have access to the data. No information will allow responses to be linked to a particular individual. Participation is strictly voluntary. It can be withdrawn at any time without affecting your rights in any way.

**Points of Contact**. It is understood that should any questions or comments arise regarding this study, the co-investigator [contact details here] should be contacted.

### **‘The SAP-C study- A feasibility study of the effectiveness of an intervention designed to improve Safety in Primary Care**

### **STATEMENT OF CONSENT (#2)**

I have read and understood the Participant Information Sheet associated with this research study. I have had the opportunity to ask questions. If I have asked questions, these have been answered to my satisfaction.

I understand that I don’t have to take part in this study and that I can opt out at any time. I understand that I don’t have to give a reason for opting out and I understand that opting out won’t affect my legal rights, future medical care or my job within the practice.

### I am aware of the potential risks of this research study.

**Storage and future use of information:**

I give my permission for information collected about me to be stored or electronically processed for the purpose of scientific research and to be used in related studies or other studies in the future but only if the research is approved by a Research Ethics Committee.

**Statement of Consent**: I have read the information provided above. I have been given the opportunity to ask questions and have had enough time to consider the information. I understand that my participation is voluntary and that I am free to withdraw this at any stage, without giving any reason, without my legal rights being affected.

### General Practitioner Name (Block Capitals):

General Practitioner Signature:

Date:

**Patient Chart Audit:** I have read the information provided regarding the patient chart audit that is to be conducted if my practice is randomly assigned to the intervention group. I wish to indicate that I am willing to conduct the patient chart audit in my practice. I understand that if I am selected to conduct this chart audit that I will receive training and guidance.

General Practitioner Signature:

**Participant Information Sheet (#3) (for General Practitioners in practices assigned to the intervention group who have indicated they are interested in conducting the in-practice patient chart audit)**

**Introduction.** You are being invited to carry out two patient chart audits as part of your participation in the intervention group of the Health Research Board funded study entitled ‘The SAP-C study- A feasibility study of the effectiveness of an intervention designed to improve Safety in Primary Care’.

**Procedures.** As part of each patient chart audit, you will be asked to review 20 randomly selected patient records from a sample of high-risk patients (> 75 years of age) using a special trigger tool that will be provided by the researchers. Prior to conducting the audits, you will receive comprehensive training in the use of this trigger tool from a member of the research team. It is anticipated that each of the chart audits will take 2-3 hours of your time. You will provide summary information to the researchers on the outcomes of this audit (i.e., number of charts reviewed, number of incidents of concern identified etc.) but the researchers will not access any patients charts or nor will they require any information on specific patients. You will be encouraged to discuss the outcomes of the patient chart audit with your colleagues and to provide feedback on the outcomes where appropriate.

**Risks.** The potential risks of participating in this chart audit are that you may determine that safety practices in your general practice are less than optimal, or that you may identify instances of patient harm. These risks are no greater than the risks associated with the chart audit and practice evaluation that should be conducted yearly by GPs to comply with the Irish College of General Practitioner’s continuing professional development guidelines. There is also a risk of a breach in confidentiality. However, all data that you provide will be coded in order to ensure that individual participants are not identifiable from the data and will be stored securely.

**Benefits.** Participation in this study may aid with the improvement of the safety practice of your general practice and may result in improved patient safety. The results of the study will also be used to inform further study of the safety climate of general practice in Ireland and may lead to improved patient outcomes nationally.

**Compensation.** Participants who complete the patient chart audit will be compensated financially, at a locum rate, for the time required to complete each audit and the time required to take part in the associated training. Participants may also claim ICGP continuing professional development credits for completing the audit as part of the requirements surrounding practice evaluation and development.

**Confidentiality.** All data collected will be confidential and will be stored securely. Data will be stored securely and participants will not be identifiable from the data.

**Voluntary Nature of the Study**. Any information that is obtained during the study will be kept confidential. Only the researchers will have access to the data. No information will allow responses to be linked to a particular individual. Participation is strictly voluntary. It can be withdrawn at any time without affecting your rights in any way.

**Points of Contact**. It is understood that should any questions or comments arise regarding this study, that the co-investigator [contact details here] should be contacted.

### ‘**The SAP-C study- A feasibility study of the effectiveness of an intervention designed to improve Safety in Primary Care**

### **STATEMENT OF CONSENT (#3)**

### I have read and understood the Participant Information Sheet associated with this research study. The information has been fully explained to me and I have been able to ask questions, all of which have been answered to my satisfaction.

I understand that I don’t have to take part in this patient chart audit and that I can opt out at any time. I understand that I don’t have to give a reason for opting out and I understand that opting out won’t affect my legal rights, future medical care or my job within the practice.

I am aware of the potential risks of this research study.

**Storage and future use of information:**

I give my permission for information collected about me to be stored or electronically processed for the purpose of scientific research and to be used in related studies or other studies in the future but only if the research is approved by a Research Ethics Committee.

**Statement of Consent**: I have read the information provided above. I have been given the opportunity to ask questions and have had enough time to consider the information. I understand that my participation is voluntary and that I am free to withdraw this at any stage, without giving any reason, without my legal rights being affected.

### General Practitioner Name (Block Capitals):

General Practitioner Signature:

Date:

**Invitation Letter (#3) (Other General Practice Staff Members - to be sent following receipt of consent from the Principal GP)**

Dear Sir/Madam,

As part of a practice registered with the Irish Primary Care Trials Network, you are being invited to participate in a Health Research Board funded study entitled ‘The SAP-C study- A feasibility study of the effectiveness of an intervention designed to improve Safety in Primary Care’.

You are being invited to participate in this research study as the principal GP(s) in your practice have indicated their willingness for the practice staff members to take part if they choose to. The enclosed participant information sheet provides comprehensive information on what participation in this study involved. Please read this information sheet carefully if you are interested in taking part. Participation is voluntary and anonymous. If you wish to take part having read the participant information sheet, please return a signed copy of the statement of consent form to the researchers.

Patient safety is a core element of the eight domains of good professional practice as outlined by the Irish Medical Council. However, medical errors remain common and can result in considerable patient harm. A large volume of work has examined interventions to improve patient safety in the hospital setting but little research has considered patient safety in primary care settings, although the majority of consultations occur in these contexts. With increasing demands on general practitioners in terms of workload, time constraints, early discharges from hospital and polypharmacy, there is a significant potential for error that must be addressed.

The purpose of this research study is to evaluate an intervention intended to improve patient safety in primary care settings. The intervention that will be implemented is adapted from the NHS Safer Primary Care Scotland model. The intervention includes the measurement of safety climate in participating general practice settings (via questionnaire distributed to all staff members) and provision of individualised feedback to participating practices. The effects of the intervention will be measured by comparing the outcomes of those experiencing the intervention to those of a control group. Practices that participate will be randomly assigned to either the intervention or control groups.

Please do not hesitate to contact [co-investigator] if you need any further information, would like to discuss this study in person, or have any questions regarding this research study. Thank you for taking the time to read this.

Kind regards,

[Investigators’ details here]

**Participant Information Sheet (#3) (Other General Practice Staff Members)**

**Introduction.** You are being invited to participate in a Health Research Board funded study entitled ‘The SAP-C study- A feasibility study of the effectiveness of an intervention designed to improve Safety in Primary Care’

**Procedures.** Participating practices will be randomly assigned to either the intervention group or control group. The activities involved differ between the groups and are described below.

*Intervention group:* If you are assigned to the intervention group, you will be asked to complete a questionnaire concerning patient safety in your practice at three timepoints (commencement of intervention, mid-intervention, and at the conclusion of the intervention). This questionnaire will take no more than 10 minutes to complete.

*Control group:* If you are assigned to the control group, you will be asked to complete a questionnaire concerning patient safety in your practice at two timepoints (commencement of intervention and at the conclusion of the intervention). This questionnaire will take no more than 10 minutes to complete.

**Risks.** The potential risks of participating in this study are that you may determine that safety practices in your general practice are less than optimal. These risks are no greater than the risks associated with the chart audit and practice evaluation that should be conducted yearly by GPs to comply with the Irish College of General Practitioner’s continuing professional development guidelines. There is also a risk of a breach in confidentiality. However, all data that you provide will be coded in order to ensure that individual participants are not identifiable from the data and will be stored securely.

**Benefits.** Participation in this study may aid with the improvement of the safety practice of your general practice and may result in improved patient safety. The results of the study will also be used to inform further study of the safety climate of general practice in Ireland and may lead to improved patient outcomes nationally.

**Compensation.** Participants who complete the questionnaire will be entered into a prize draw to win one of several 250e vouchers at each questionnaire completion timepoint.

**Confidentiality.** All data collected will be confidential and will be stored securely. Data will be stored securely and participants will not be identifiable from the data.

**Voluntary Nature of the Study**. Any information that is obtained during the study will be kept confidential. Only the researchers will have access to the data. No information will allow responses to be linked to a particular individual. Participation is strictly voluntary. It can be withdrawn at any time without affecting your rights in any way.

**Points of Contact**. It is understood that should any questions or comments arise regarding this study, that co-investigator [contact details here] should be contacted.

### **‘The SAP-C study- A feasibility study of the effectiveness of an intervention designed to improve Safety in Primary Care**

### **STATEMENT OF CONSENT (#3)**

I have read and understood the Participant Information Sheet associated with this research study. I have had the opportunity to ask questions. If I have asked questions, these have been answered to my satisfaction.

I understand that I don’t have to take part in this study and that I can opt out at any time. I understand that I don’t have to give a reason for opting out and I understand that opting out won’t affect my legal rights, future medical care or my job within the practice.

### I am aware of the potential risks of this research study.

**Storage and future use of information:**

I give my permission for information collected about me to be stored or electronically processed for the purpose of scientific research and to be used in related studies or other studies in the future but only if the research is approved by a Research Ethics Committee.

**Statement of Consent**: I have read the information provided above. I have been given the opportunity to ask questions and have had enough time to consider the information. I understand that my participation is voluntary and that I am free to withdraw this at any stage, without giving any reason, without my legal rights being affected.

### Participant Name (Block Capitals):

Participant Signature:

Date:
